# Early Robotic-Assisted Laparoscopic Pyeloplasty for Infants Under 3 Months With Severe Ureteropelvic Junction Obstruction

**DOI:** 10.3389/fped.2021.590865

**Published:** 2021-03-10

**Authors:** Pin Li, Huixia Zhou, Hualin Cao, Tao Guo, Weiwei Zhu, Yang Zhao, Tian Tao, Xiaoguang Zhou, Lifei Ma, Yunjie Yang, Zhichun Feng

**Affiliations:** ^1^Department of Pediatric Urology, Bayi Children's Hospital, Affiliated of the Seventh Medical Center of People's Liberation Army General Hospital, Beijing, China; ^2^The Second School of Clinical Medicine, Southern Medical University, Guangzhou, China; ^3^Department of Urology, Nan Xi Shan Hospital of Guangxi Zhuang Autonomous Region, Guilin, China; ^4^Medical School of Chinese People's Liberation Army, Beijing, China; ^5^Department of Pediatrics, The Third Medical Center of People's Liberation Army General Hospital, Beijing, China; ^6^Department of Urology, The Affiliated Nanhai Hospital of the Southern Medical University, Foshan, China

**Keywords:** robotic-assisted laparoscopic pyeloplasty, infant, hydronephrosis, ureteropelvic junction obstruction, RALP

## Abstract

**Objective:** To present our primary experience of robotic-assisted laparoscopic pyeloplasty (RALP) for severe ureteropelvis junction obstruction (UPJO) infants under 3 months.

**Methods:** We performed a retrospective study of 9 infants under 3 months who underwent RALP for severe UPJO between April 2017 and March 2019 in our center. The severe UPJO was defined as infants with severe hydronephrosis (Society of Fetal Urology grades III or IV, anteroposterior diameter >3 cm or split renal function <40% or T 1/2 >20 min) involving bilateral, solitary kidney, or contralateral renal hypoplasia UPJO at the same time. All clinical, perioperative, and postoperative information was collected.

**Results:** There were four bilateral UPJO cases, two solitary kidney UPJO cases and three unilateral UPJO with contralateral renal hypoplasia cases included. One single surgeon performed RALP on all of the infants. The mean age of the infants was 1.62 ± 0.54 months. The mean operative time was 109.55 ± 10.47 min. The mean estimated blood loss was 19.29 ± 3.19 ml, and the mean length of hospital stay was 5.57 ± 0.73 days. According to the ultrasonography results, all patients had a significant recovery of renal function at 12 months after the operation.

**Conclusions:** To maximize the protection of renal function, early RALP is a safe and feasible option for the treatment of severe UPJO in infants under 3 months.

## Introduction

Ureteropelvic junction obstruction (UPJO) is one of the major causes of infant hydronephrosis (1). The management of UPJO has evolved from open pyeloplasty (OP), laparoscopic pyeloplasty (LP), and robotic-assisted laparoscopic pyeloplasty (RALP) (2, 3). Well-established evidence has demonstrated that LP or RALP not only has success rates equal to those of OP but also has the advantages of minimal invasiveness, better cosmesis, less post-operative pain, decreased length of hospital stay, and early recovery^1^ (4, 5). In general, the management of hydronephrosis included conservative observation and surgical invention. The clinical decision making usually depends on the rate of hydronephrosis severity. There is still no consensus on the optimal intervention time to perform the surgery, however; whether through conservative or surgical treatment, the ultimate goal is to maximally protect renal function.

Severe UPJO generally refers to bilateral UPJO, a solitary kidney with UPJO, or UPJO with contralateral renal dysplasia. In these complex situations, the selection of conservative observation, conservative nephrostomy, or early minimal invasive pyeloplasty is a problem, especially for very young children under 3 months of age. It is widely acknowledged that pyeloplasty for an infant under 1 year of age or under 10 kg of weight is a challenging procedure that requires more elaborate techniques to decrease the number of complications and lessen operating time to reduce the negative effect of anesthesia (6, 7). In this retrospective study, we summarize our initial experience with conducting RALP on nine severe UPJO infants under 3 months of age.

## Methods and Materials

### Patients

**Figure 1:**
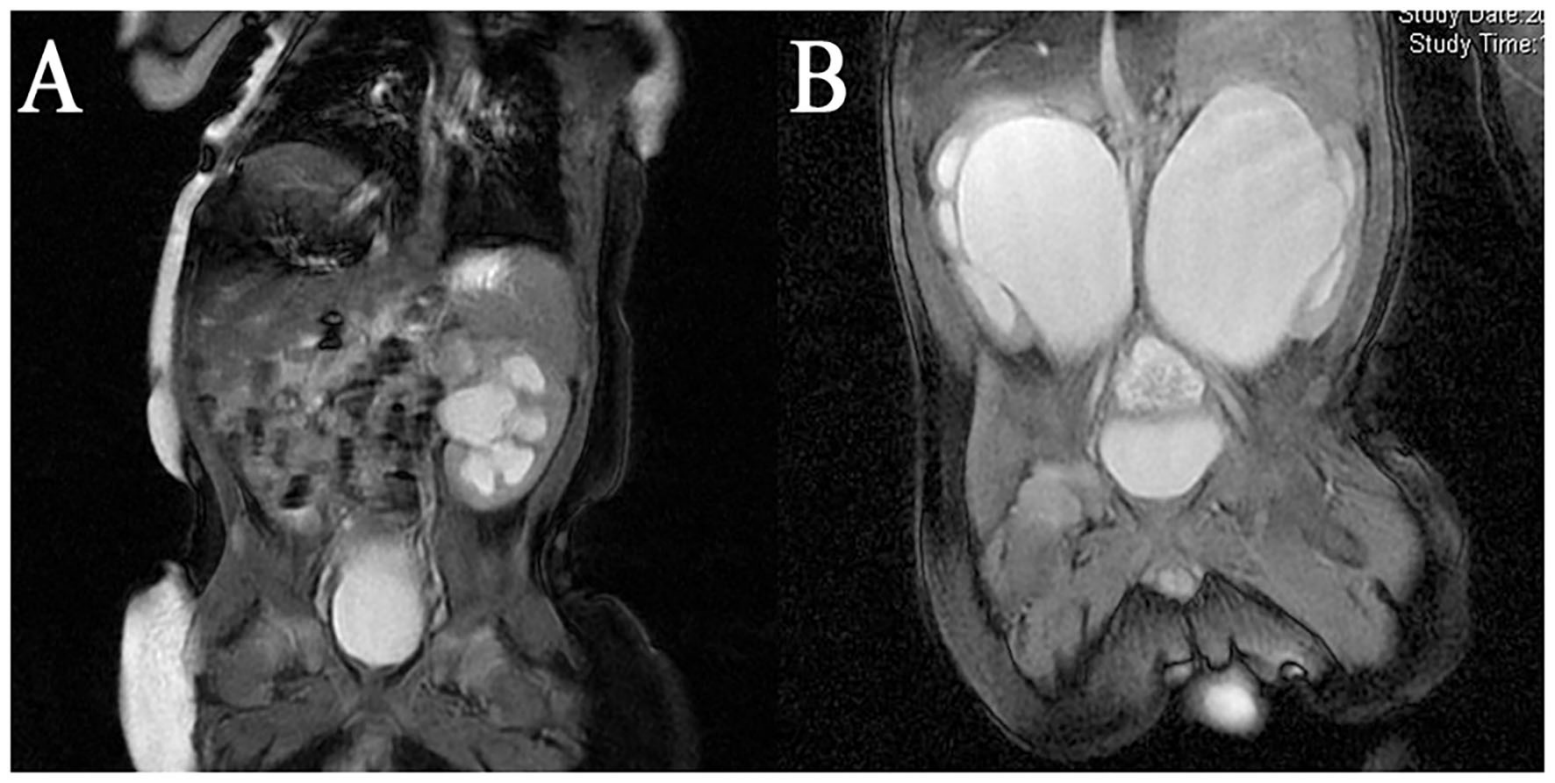
MRI result of a **(A)** Unilateral UPJO with contralateral renal dysplasia; **(B)** bilateral severe UPJO.

Nine infants 0.8–2.6 months old (mean age 1.62 months) presented with severe UPJO confirmed by ultrasonography screening and were referred to our center from April 2017 to March 2019. The inclusion criteria of this study included age <3 months, severe hydronephrosis defined as grade III and IV dilation as defined by the Society for Fetal Urology (SFU), anteroposterior diameter (APD) more than 3 cm, impaired split renal function <40%, along with one of the following three conditions: bilateral UPJO, solitary kidney UPJO, or unilateral severe UPJO with contralateral renal dysplasia. Exclusion criteria were UPJO with megaureters, vesicoureteral reflux, posterior urethral valve, or the existence of other structural anomalies. The diagnosis was based on ultrasonography, magnetic resonance urography (MRU) (Figure 1), voiding cystourethrography (VCUG), radionuclide, and 99mTc-mercaptoacetyltriglycine (MAG3) diuretic renography results. Perioperative demographic information was also recorded. All patients underwent robotic-assisted laparoscopic pyeloplasty (RALP) with one single surgeon. The Clavien-Dindo classification system was used to evaluate the postoperative complications. This study was undertaken with the approval of the Seventh Medical Center of PLA General Hospital Institutional Ethics Committee. All patients' parents have signed the written consent forms.

### Surgical Technique

**Figure 2:**
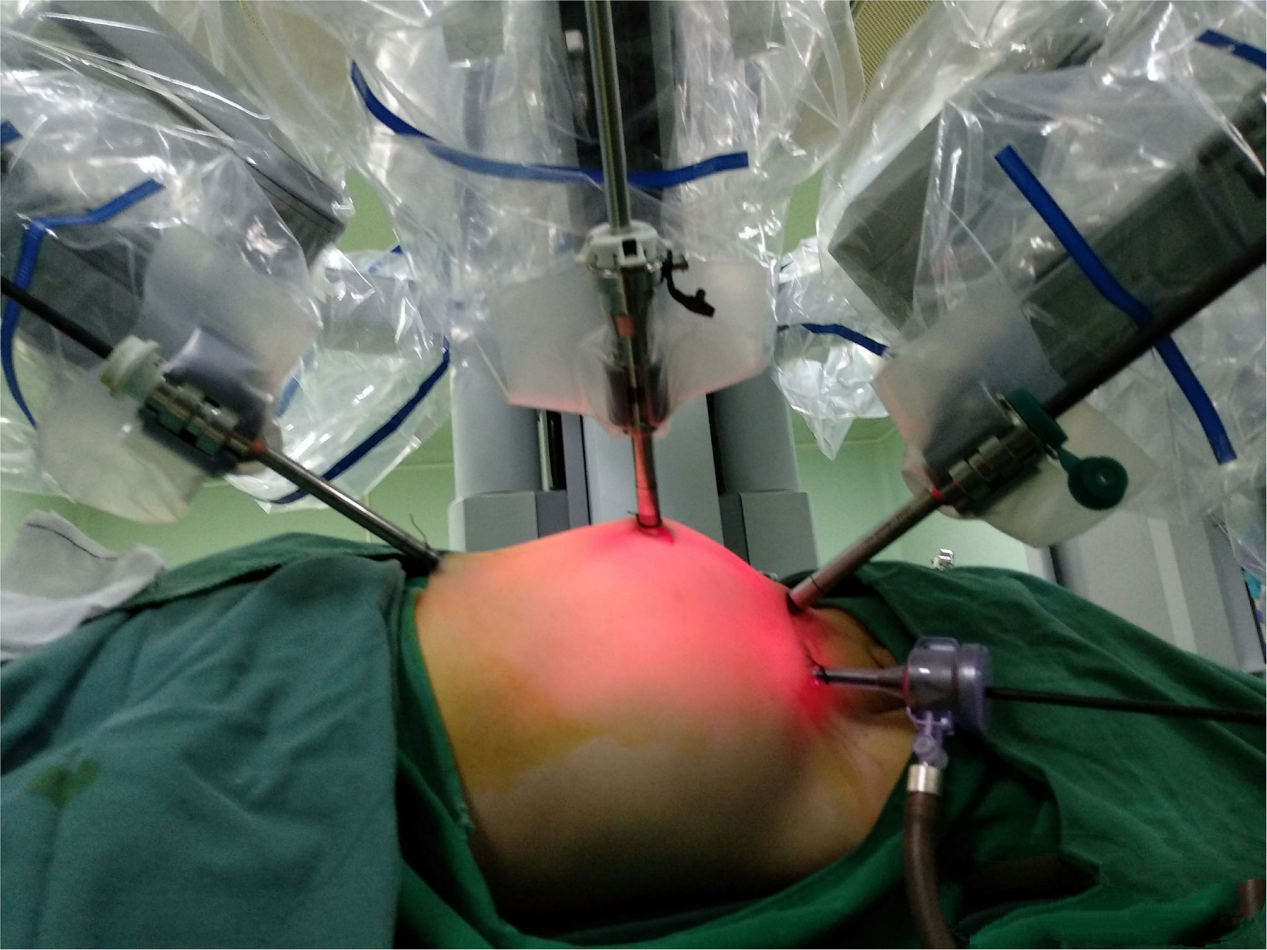
Trocar position appearance.

After routine preoperative preparation and anesthesia, pnuemoperitoneum was established and maintained at 6–8 mmHg pressure. All ports were placed under direct vision included one 8.5 mm camera trocar, one 8-mm trocar and one 5-mm trocar. One or two additional assistant 3-mm trocars were placed at the lateral 3 cm of the midpoint of the Pfannenstiel line, to improve the efficient of the suture (Figure 2). For left side cases, the transmesenteric approach was adopted while the dilated renal pelvis was located at the inside of the descending colon. For right side cases, we selected the paracolic sulci approach. Then we carefully dissected the proximal ureter and renal pelvis while preserving the ureteral blood supply. The pelvis was cut above the obstruction tissue and trimmed by a percutaneous hitch stich to stabilize it and facilitated the anastomosis. After spatulated the distal ureter after excision of the obstruction segment, we sutured the lowest point of the aperistaltic ureteral segment and the pelvis end with a running 6-0 PDS-II. Then the posterior wall of the ureter was closed through continuous suture. Before the anterior anastomoses were started with a second running 6-0 PDS II suture, a double-J ureteral stent (COOK, USI-512, Ireland) was placed antegrade. At last, we closed the mesenterium or peritoneum with a 5-0 absorbing suture. For the bilateral pyeloplasty infants, we performed one sided RALP and nephrostomy for the other side. After 1 week interval, we performed RALP for the contralateral UPJO in the same way.

### Postoperative Management

The infants restarted general oral feeding after they had recovered from anesthesia. The double-J stent was removed under general anesthesia 6–8 weeks after the operation by cystoscopy. Ultrasonography, radionuclide, and diuretic renography examinations were repeated the 6th and 12th months after surgery.

### Statistical Analysis

Continuous data were presented as the mean ± STD and range. Functional outcomes were compared using the Student *t*-test or chi-square test. All statistical analyses were performed in the R software environment (version 3.6.3; http://r-project.org/), and *p* < 0.05 was considered significant in all statistical analyses.

## Results

The baseline clinical data of the nine infants were shown in Table 1. All operations were performed successfully without conversion to open surgery. No serious intraoperative complication happened. The perioperative findings were summarized in Table 2. Two patients with postoperative infection (Clavien-Dindo Grade II Complications) were managed conservatively by intravenous antibiotics. No patient suffered Clavien III or IV complications. The mean time for Foley catheter removal was 1.86 ± 0.64 days.

**Table 1:** Patient characteristics.

**Description**	**No**.
Patient	9
Age at surgery, month, mean ± SD (range)	1.62 ± 0.54 (0.8–2.6)
Gender, No. male/female	6/3
Diagnosis	
Solitary kidney with UPJO	2
UPJO with contralateral renal dysplasia	3
Bilateral UPJO	4
APD (mm), mean ± SD(range)	4.06 ± 0.73(3.4–5.3)
SFU Grade III/IV	4/9
Split renal function	0.36 ± 0.04
Renography T1/2 >20 min	8

**Table 2:** Perioperative outcomes.

**Description**	**No**.
Estimated blood loss	19.29 ± 3.19(15–30)
Operation time	109.55 ± 10.47(92–138)
Conversion to open surgery	0
Foley catheter indwelling days	1.86 ± 0.64
Length of hospital stay	5.57 ± 0.73
Complications Clavien-Dindo	
I and II	2
III and IV	0

According to the follow-up data listed in Table 3, the renal pelvis APD decreased to 0.97 ± 0.16 cm in the 6th month after surgery, which was significantly smaller than perioperative APD (*p* < 0.01). Radionuclide renography results showed that the split renal function had a great improvement in 6 months and slightly increased in 12 months. Diuretic renography revealed that 8 out of 9 patients have a T 1/2 time <10 min in the 6th month after surgery. In the 12th month examination, all of the 9 patients' T 1/2 times were <10 min.

**Table 3:** Preoperative and follow-up characteristics.

**Description**	**Pre-operation**	**6th month**	**12th month**	***p*-value**
APD (mm)	4.06 ± 0.73	0.97 ± 0.16	0.86 ± 0.12	<0.01
Split renal function	0.36 ± 0.04	0.53 ± 0.05	0.58 ± 0.04	<0.01
Renography T1/2 <10 min	0	8	9	<0.01

## Discussion

Open dismembered pyeloplasty has been the gold standard treatment for UPJO for decades with overall success rates of more than 90% (8). Since first reported in 1993, laparoscopic pyeloplasty has been demonstrated as a safe and effective treatment for UPJO (9). Two years later, pediatric laparoscopic pyeloplasty was introduced by Peters et al. (10). While limited by the small space for instrument movement and trocar placement, the use of laparoscopic and robotic-assisted laparoscopic is well-described (11, 12). Recently, more and more literature has proved that laparoscopic pyeloplasty or robotic-assisted laparoscopic pyeloplasty has not only the same success rate as open pyeloplasty, but also shorter hospitalization stay, faster recovery time, and better cosmetic appearance (13–16).

Meanwhile, the management of hydronephrosis in children has greatly changed during the last 20 years. In the 1990s, Ransley et al. (17) reported that early pyeloplasty may not be of greater benefit than observed or delayed surgery. After radiological imaging studies had become available for clinical evaluation, the value of split renal function and T1/2 was greatly improved for deciding the optimal time for surgical treatment (18). According to the results of a study conducted by Onen et al., (19) they only recommended surgical intervention for renal deterioration (decreased split renal function or progressive hydronephrosis). However, Tabari et al. (20) revealed that early pyeloplasty could benefit infants <1 year old suffering from severe but asymptomatic hydronephrosis better than conservative management through a prospective interventional study. In their study, they compared the functional outcomes of open pyeloplasty on a group of infants and conservative management of infants. They found that the group of infants who had early surgery have lower SFU grade and larger cortical thickness than the conservative group. According to the EAU Guidelines 2020, increased APD, SFU grade III or IV, split renal function <40%, or decrease >10% in follow-up and poor drainage function could be indications for asymptomatic UPJO.

For infants under 1 year old or even under 3 months, there are numerous challenges for surgical intervention so that whether to perform surgery is controversial. In 2006, Kutikov et al. (21) reported that transperitoneal laparoscopic pyeloplasty for UPJO in eight infants under 6 months old is technically possible. Zamfir Snykers et al. (22) also draw a similar conclusion in their research. Simforoosh et al. (14) compared the surgical outcomes of standard and minilaparoscopic pyeloplasty in children younger than 1 year of age. They believed that both of these approaches had the same effect while the minilaparoscopic technique could be more cosmetically pleasing and less invasive (14). In a retrospectively study, Turner et al. (23) assessed the effect of laparoscopic pyeloplasty performed in 29 infants 2–11 months old. Their experience revealed a success rate with minimal morbidity (23). In a multi-institutional trial, Daniel et al. (16) collected perioperative data of 60 patients underwent RALP by six surgeons and described an excellence success rate and a low complication rate in this cohort. Shukla et al. (24) summarized their experience about RALP and compared outcomes between infants aged <1 year and older children. They found that there were no significant differences in length of hospital stay and complications or failure rates in infants compared to older children, and they called for the adaptation of RALP for the entire pediatric patient population. Andolfi et al. (6) conducted a systematic review to compare whether RALP is superior to conventional LP. They selected 19 original articles and 5 meta-analyses and concluded that RALP could decrease operative times, shorten the length of hospital stay, and reduce the complication rates while having the same success rates comparable to LP.

Conventional laparoscopy has a significant learning curve and is technically challenging for many surgeons compared to robot-assisted laparoscopy. Undoubtedly, the robotic-assisted technique can facilitate a shorter learning curve and act as a bridge between the open and endoscopic approaches. In these years, pediatric RALP has become a viable minimally invasive surgical option for UPJO children with some reports on its efficacy, safety, and cosmetic effect (15, 25, 26). Our team has also presented our experiences of transumbilical multi-stab laparoscopic pyeloplasty for infants younger than 3 months. On this basis, we performed RALP for these severe hydronephrosis patients under 3 months in this cohort.

This study included nine infants (thirteen sides) ranging from 0.8 to 2.6 months old who underwent transperitoneal RALP. All of the patients were diagnosed prenatally and had regular examinations after birth. As the hydronephrosis lasted and became even worse, we decided to intervene early with these patients because of our previous experience with the children who had undergone RALP. For the infants who were sensitive to the CO_2_ pressure, we usually selected 6–8 mmHg to establish the existence of pneumoperitoneum. To expand the operating space as much as possible, we lifted and fixed robotic arm numbers 1 and 2. We have also explored several port positions for infants and finally selected the strategy described in this article as it could provide the most operating space and the least skin wounds. To reduce the incidence of anastomosis obstruction and improve success rates, several techniques were applied in RALP, including the way to identify the axis of renal calyx as the kidney axis and started the anastomosis at the lowest point of the renal pelvis and ureter, which was also described in our previously published literature (27). For the bilateral UPJO cases, we performed two RALPs at one-week intervals, but not side by side, as bilateral RALP had longer operating time and higher stent blockage risk. During the hospitalizations, no anesthesia complications were observed. Our clinical experience indicated that these techniques are important to facilitate RALP and improve success rates and decrease postoperative complications (27). According to follow-up data from the 6th and 12th months after operation, the primary outcomes were positive. T1/2 results showed no obstruction of the ureter after 12 months. The cosmetic appearance was also satisfactory although in our study the quantitative evaluation was not. Compared with our previous published study about our early experience of using LP for infants younger than 3 months (28), RALP (including docking time) has a longer operation time (109.55 vs. 75 min), same length of stay (5.57 vs. 6 d) and the same success rate. LP has a litter advantage on the cosmetic effect, but the learning curve of RALP is significantly decreased.

The limitations of this study include its retrospective nature, lack of randomization and design with no control group, small patient sample size, use of a single center, the lack of more than one surgeon with experience with RALP, and the focus only on primary outcomes within 1 year. These factors limit us from drawing more conclusions on the management of severe hydronephrosis. Despite the existence of these limitations, we believe that our study provides new insight into the application of the robotic technique in infant surgery. It confirms that RALP has the advantage of being minimally invasive and could be used to protect the renal function of severe UPJO patients under 3 months as early as possible.

## Conclusion

Early RALP is a safe and feasible option for the treatment of severe UPJO infants under 3 months. However, further controlled prospective study is still necessary to determine the ultimate role of RALP in the management of young infants with UPJO.

## Data Availability Statement

The raw data supporting the conclusions of this article will be made available by the authors, without undue reservation.

## Ethics Statement

The studies involving human participants were reviewed and approved by the Seventh Medical Center of PLA General Hospital Institutional Ethics Committee. Written informed consent to participate in this study was provided by the participants' legal guardian/next of kin.

## Author Contributions

Conceptualization: PL and HZ. Data curation and Investigation: PL and HC. Formal analysis: PL and TG. Funding acquisition and Project administration: HZ. Methodology: LM. Resources: TT, XZ, and LM. Software: WZ and YZ. Supervision: ZF and HZ. Validation: YY. Visualization: PL, HC, and TG. Writing—original draft: PL and HC. Writing—review and editing: HZ. All authors contributed to the article and approved the submitted version.

## Conflict of Interest

The authors declare that the research was conducted in the absence of any commercial or financial relationships that could be construed as a potential conflict of interest.

## Footnotes

^1^The laparoscopic pyeloplasty: is there a role in the age of robotics? Accessed May 6, 2020. https://www.ncbi.nlm.nih.gov/pubmed/25455171
